# Towards seeing the visual impairments in Parkinson’s disease: protocol for a multicentre observational, cross-sectional study

**DOI:** 10.1186/s12883-019-1365-8

**Published:** 2019-06-25

**Authors:** Carlijn D. J. M. Borm, Mario Werkmann, Femke Visser, Marina Peball, Diana Putz, Klaus Seppi, Werner Poewe, Irene C. Notting, Annemarie Vlaar, Thomas Theelen, Carel Hoyng, Bastiaan R. Bloem, Nienke M. de Vries

**Affiliations:** 1 Department of Neurology, Parkinson Center Nijmegen (ParC) Nijmegen, Donders institute for Brain, Cognition and Behaviour, Radboud University Medical Centre, PO Box 9101, 6500 HB Nijmegen, The Netherlands; 2 Radboud University Medical Centre, Department of Ophthalmology, Nijmegen, The Netherlands; 3 Department of Neurology, Medical University Innsbruck, Innsbruck, Austria; 4 Department of ophthalmology, Medical University Innsbruck, Innsbruck, Austria; 5 Department of Neurology, Onze Lieve Vrouw Gasthuis (OLVG), Amsterdam, The Netherlands; 6 Department of Ophthalmology, Leiden University Medical Centre, Leiden, The Netherlands

**Keywords:** Parkinson’s disease, Ophthalmology, Eye, Visual impairment, Non-motor symptoms, Screening questionnaire, Survey

## Abstract

**Background:**

Visual disorders are common in Parkinson’s disease (PD) but their exact frequency and severity are unknown. Good visual functioning is crucial for patients with PD, because of their need to compensate for loss of automated motor control and their postural instability, forcing patients to guide their movements visually. Here, we describe the study design of a cross-sectional, multi-centre study aiming to: (1) validate the Visual Impairment screening questionnaire (VIPD-Q, which aims to identify PD patients who should be referred to an ophthalmologist for further assessment); (2) study the prevalence of visual disorders in PD; (3) study the severity and clinical impact of different types of visual disorders in PD; and (4) explore treatment options for ophthalmologic disorders in PD, as a basis for future guideline development.

**Methods:**

This study consists of two phases. In phase one, 750 PD patients and 250 healthy controls will be recruited to complete the VIPD-Q. In phase two, a subgroup of responders (*n* = 100) (with the highest and lowest scores on the VIPD-Q) will be invited for an extensive neurological and ophthalmological assessment. The in-depth ophthalmologic examination will serve as the “gold standard” for validating the VIPD-Q. Moreover, these assessments will be used to study associations between visual disorders and clinical presentation, in order to gain more insight in their clinical impact.

**Discussion:**

Our study will heighten the awareness of visual problems in PD and offers a robust starting point to systematically approach this subject. In current daily practice, the association between visual problems and PD is far from obvious to both patients and clinicians. Consequently, patients may not adequately report visual problems themselves, while clinicians miss potentially treatable visual disorders. Routinely asking patients to complete a simple screening questionnaire could be an easy solution leading to timely identification of visual problems, tailored treatment, restored mobility, greater independence and improved quality of life.

**Trial registration:**

Dutch Trial Registration, NL7421, Registered on 4 December 2018 – Retrospectively registered.

**Electronic supplementary material:**

The online version of this article (10.1186/s12883-019-1365-8) contains supplementary material, which is available to authorized users.

## Background

Parkinson’s disease (PD) is the second most common neurodegenerative disease in the developed world. The disease is characterized by a broad range of motor and non-motor symptoms [1]. Non-motor-symptoms include widely recognised examples such as gastrointestinal complaints, cognitive decline or autonomic dysfunction, but also less well appreciated ones such as visual impairment [2]. Visual problems can range from blurred vision, diplopia, reduced colour and contrast vision to visual field deficits, and sore, red, or fatigued eyes. Visual disorders in PD can be caused by different pathological mechanisms like retinal dopamine depletion or decreased dopaminergic innervation of the visual cortex [3, 4].

Almost 80% of visual impairments are treatable or preventable. But to achieve this, timely recognition is obviously pivotal [5]. However, in the field of PD, the presence of visual problems and the resultant visual impairment has remained severely under-recognized, both in research and in clinical practice. This is surprising, since the impact of visual problems is particularly vexing for patients with PD, because of their need to compensate for loss of automated motor control and their postural instability, forcing patients to guide their movements externally (and this include visual guidance) [6]. For example, visual cues such as stripes on the floor are commonly used in clinical practice to overcome freezing of gait [2, 7, 8]. Not being able to see these visual cues adequately may have an immediate impact on functioning in daily life. Indeed, visual disorders combined with postural instability and gait disability can increase the risk of falls and fall-related injuries such as hip fractures and head injuries [9, 10]. Other obvious consequences of visual deficits in PD include problems with driving and reading. Taken together, disorders of vision can lead to reduced physical activity, greater dependence, disability, injuries and reduced quality of life [11–13]. Yet, the exact frequency, type and severity of visual problems are unknown. The few prior studies on this subject showed inconclusive results, in part due to small samples or lack of a complete ophthalmologic examination [14–16]. Since many of these disorders can be treated effectively, educating patients and doctors about them can help to improve quality of life of patients with PD.

The current multicentre observational, cross-sectional VIP study: “Visual Impairment in Parkinson’s disease” is designed to promote knowledge about and recognition of symptoms, signs and causes of disturbed vision in people with PD. Our specific goals are to: (1) validate a screening questionnaire that aims to identify PD patients who should be referred to an ophthalmologist for further assessment; (2) determine the prevalence and severity of visual problems in PD; (3) study the impact of ophthalmologic disorders on clinical functioning (in particular on gait and balance control); and (4) explore treatment options for ophthalmologic disorders in PD, as a basis for future guideline development.

## Methods/design

**Fig. 1:**
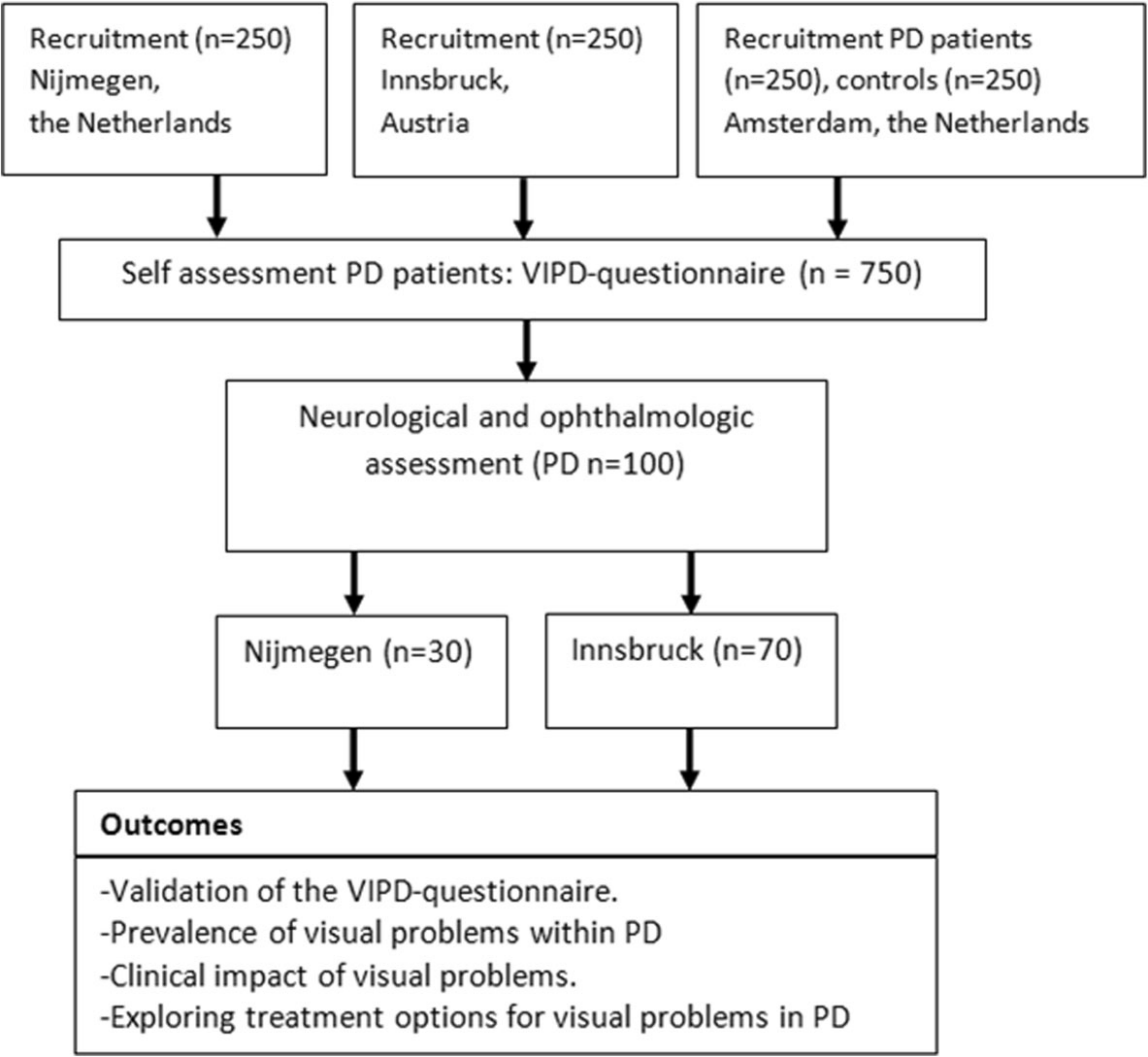
Flowchart of the study design. Here we present our two-phase study. In phase 1, at least 750 PD patients and 250 healthy controls will complete the Visual impairment in Parkinson’s Disease Questionnaire (VIPD-Q). In phase 2, a subgroup of responders (*n* = 100) will be invited for an extensive neurological and ophthalmological assessment

Here we present our two-phase study, shown in Fig. 1. In phase 1, we aim to have the Visual impairment in Parkinson’s Disease Questionnaire (VIPD-Q) completed by at least 750 PD patients and 250 healthy controls. In phase 2, a subgroup of responders (*n* = 100) will be invited for an extensive neurological and ophthalmological assessment. The study is performed at three study sites (Radboud University Nijmegen Medical Centre, the Netherlands; the Medical University Innsbruck, Austria; and The OLVG hospital Amsterdam, the Netherlands). Each centre obtained local ethical approval (NL58535.091.16;AN2016–0181).The study will be performed in accordance with the Declaration of Helsinki. All participants have to give written informed consent prior to participation. This study is registered in The Dutch trial register: NTR 7663.

### Phase 1: screening questionnaire

The VIPD-Q was developed by neurologists and ophthalmologists to detect a broad range of ophthalmologic disorders in PD patients. It is based on an extensive literature study, previous questionnaires, and common and disabling visual disorders in both PD patients and healthy older people [2, 11, 13, 15, 17–34]. The questionnaire includes 22 questions on visual problems, plus a standard set of demographic data (Additional file 1: Appendix 1). Answers are given on a 4-point Likert scale ranging from “never have problems” to “daily problems”, without possibility to give a neutral answer. As a second step, we grouped the questions according to the anatomical location of a visual disorder. These domains were agreed upon by a consensus procedure involving three independent ophthalmologists (one from each study site). They were asked to categorize the questions in four domains (Table 1: ocular surface, oculomotor, intra-ocular and optic nerve). Consensus was reached during a meeting with the three ophthalmologists. Six questions could not be categorized in one domain, but rather indicate the involvement of more than one anatomical domain. The VIPD-Q is designed as a patient self-scoring instrument and takes about 20 min to complete.

**Table 1:** Domains and corresponding questions with possible diagnosis

Domains	Issues	Possible diagnosis	Question
Ocular surface	lacrimal fluid, eyelids, conjunctiva, cornea	dry eye disease, blepharitis, blepharospasm, conjunctivitis, low eye blink rate,	1: I have blurry vision, when I read or work on a computer. 2: I have a burning sensation or gritty feeling in my eyes. 3: I have mucus/slime or particles in my eyes or eyelids. 4: I have watery eyes.
Intra-ocular	lens, uvea, macula, retina	diabetic retinopathy, macular degeneration, retinal detachment, hypertensive retinopathy, micro-vascular retinopathy, cataract, refraction errors, glaucoma	5: When I read, some letters disappear. 6: Lines that should be straight, appear to be wavy or blurred. 7: I won’t go out alone in the evening or at night because my night vision is insufficient. 8: When I drive at night, the oncoming headlights cause more glare than before.
Oculomotor	eye movements, binocular cooperation	strabismus, convergence insufficiency, 3rd, 4th and 6th nerve palsies, nystagmus, gaze abnormality	9: Quick movements are hard to follow with my eyes. 10: I have double vision. 11: I can read better with one eye closed. 12: I have trouble with depth perception. I find it hard to say which one of two objects is closer.
Optic nerve	optic nerve disease	opticopathy, glaucoma (Mimics: structural lesion, hemianopsia)	13: Colours seem to be paler than before. 14: I can’t read plain text on a coloured or grey background. 15: I run into objects or people or feel that parts of my visual field are missing. 16. I have problems with rapid changes of light intensity. (For example, driving through a tunnel).

The Visual Impairment in Parkinson’s Questionnaire (VIPD-Q) has been divided in 4 domains as shown above. These domains were agreed upon by a consensus procedure involving three independent ophthalmologists (one from each study site). Six questions of the questionnaire could not be categorized in one domain, but rather indicate the involvement of more than one anatomical domain

The VIPD-Q is both available on paper and online (in English, German and Dutch). We will distribute the questionnaire using different strategies: (1) PD patients visiting the neurologic outpatient clinics at the three study sites (complete the questionnaire on site); (2) PD patients receiving care in one of the participating centres (will receive the questionnaire via post); and (3) PD patients interested in research, having registered on a platform for research and innovation in PD: www.parkinsonnext.nl (will receive the online questionnaire through e-mail). Healthy controls will be recruited by asking partners and family members of participating patients to also complete the questionnaire.

### Phase 2: neurological and ophthalmological assessment

#### Patient collective and recruitment

From the responders of phase 1, a sample of 100 patients will be invited for a full clinical and ophthalmological assessment. Patients will be selected based on their scores on the VIPD-Q aiming to include 70 patients with and 30 patients without visual impairment, which is operationally defined as patients scoring the highest 30% or the lowest 30% on the screening questionnaire, respectively. Due to personnel capacity, a sample of 30 patients in the Netherlands (10 in the lowest 30% and 20 in the highest 30%) and 70 patients in Austria (20 in the lowest 30% and 50 in the highest 30%) will be selected and invited for the clinical and ophthalmological assessment. The OLVG hospital only participated in phase 1 of this study. Inclusion and exclusion criteria are described in Table 2. Selection will start after at least 250 questionnaires have been completed. (See Fig. 1).

**Table 2:** Inclusion and exclusion criteria

Inclusion criteria	Exclusion criteria
Diagnosis of PD according to the UKPDSBB criteria	Hoehn and Yahr Parkinson’s staging score ≥ 4
The patient must be able and willing to give written informed consent	Secondary cause of parkinsonism as detected by history (e.g. drug-induced parkinsonism)
The patient must be willing to participate in all study related activities and visits	Secondary cause of parkinsonism as detected by investigation (e.g. vascular parkinsonism as detected by neuroimaging)
Age of onset Parkinson’s disease > 30 years	Dementia according to DSM-IV
Stable doses of Parkinson medications ≥4 weeks	Major depressive disorder according to DSM-IV
Current age ≥ 60 years	Psychotic disorder(s) according to DSM-IV
	Prior brain surgery (except deep brain stimulation)
	Previous eye surgery (except phacoemulsification for cataract and artificial lenses)
	Blindness in 1 eye
	Medication that influences normal visual function other than PD medication. (Detailed information, please see Additional file 2: Appendix 2)
	Systemic diseases that may cause eye problems (HIV, DM type I, type II if the patient had ophthalmologic therapy and/or abnormalities at last screening)
	Neurodegenerative diseases other than Parkinson’s disease
	History of lesions near the optic chiasm or occipital cortex
	Migraine

Inclusion and exclusion criteria of the Visual Impairment in Parkinson’s Disease study

Abbreviations: *UKPDSBB* United Kingdom Parkinson’s Disease Soeciety Brain Bank, *DSM-IV* Diagnostic and Statistical Manual of Mental Disorders IV, *HIV* human immunodeficiency virus, *DM type I* Diabetes Mellitus type I

#### Procedures and assessments

All assessments will be performed during regular medication use, preferably in an “ON-state”. The assessment will start with obtaining demographics and medical history. The neurological and ophthalmological assessments consist of an extensive test battery (Table 3).

**Table 3:** List of assessments

Outcome	Ophthalmological Assessment
	*Subjective ophthalmological assessment*
Visual acuity	ETDRS logMAR
Reading speed, near visual acuity	Radner Reading Chart
Visual fields	Humphrey field analyser 30–2, Amsler grid
Contrast vision	Low-contrast letter charts (Pelli-Robson),
Colour vision	Ishihara plates/Farnsworth Munsell hue test (desaturated 15D)
Ocular motility	Eye follow movements, Cover test 30 cm and 6 m, Prism test (15 diopter), Eye-Tracking
Facial emotion recognition	Emotion Recognition Inventory (ERI)
Visual function	VFQ-25, Visual impairment screening questionnaire
	*Objective ophthalmological assessment*
Lens opacity	Slit lamp examination
Fundus appearance	Color Fundusphotography (7-fields)
Corneal thickness	Pachymetry
Intraocular pressure	(Goldmann) tonometry
Tear production	Schirmer’s test, TearFilmBreakUpTime, eye blink rate
Anatomical changes of the retina including thickness	Spectral-domain OCT (Heidelberg)
Retinal micro vascular changes	Angio-OCT (Heidelberg)
Outcome	Neurological assessment
PD-related disability and impairment	MDS-UPDRS, Schwab & England activities of Daily living, NMSS, PDQ-39.
Gait and Balance	Timed Up and Go test, Single Leg Stance test, Gait Speed (4 m walking), Dual tasking, Freezing of Gait, Tandem gait
Hand-eye coordination	9 Hole Pegboard test
Cognitive function	MoCA, CLOX
Mood	GDS

List of study protocol assessments, divided in ophthalmological and neurological assessment

Abbreviations: *EDTRS* Early Treatment of Diabetic Retinopathy Study, *D* diopter, *OCT* optical coherence tomography, *NMSS* non-motor symptoms assessment scale, *PDQ-39* Parkinson’s Disease Questionnaire, MoCA [47], Montreal Cognitive Assessment, CLOX, clock drawing test [48], GDS, Geriatric depression scale [49], VFQ-25, visual functioquestionnaire [72, 73]

#### Neurological examination

PD symptoms and severity including non-motor symptoms will be measured using the Movement Disorders Society Unified Parkinson’s disease Rating Scale (MDS-UPDRS) [35]. The non-motor symptoms scale (NMSS) [36] and the Parkinson’s disease questionnaire-39 (PDQ-39- quality of life) [37] will be used to assess the full spectrum of PD symptoms. Hand-eye coordination and manual dexterity will be tested using the nine-hole peg test [38]. Mood and cognition will be assessed by the Montreal cognitive assessment (MoCA), the CLOX II test and the geriatric depression scale (GDS).

#### Gait and balance

Performance tests measuring different aspects of gait and balance will be performed. Walking velocity will be tested under single and dual-tasking conditions [39, 40]. The patients will be instructed to walk a distance of 4 m at self-preferred speed. The required number of steps will be reported as outcome. The Timed-Up-and-Go-test (TUG) measures the basic functional mobility [41]. Freezing of Gait (FOG) is assessed by letting the patient make a 360° turn with small steps (approximately 25% of their own preferred step length) and walking fast with short steps [42]. To test axial symptoms, tandem gait at a self-preferred speed is recorded for at least 10 consecutive steps without a visual guiding line [43]. The Single Leg Stance test (SLS) will be used to test static balance and postural instability [43–46]. Finally, postural stability will be recorded by the Pull-Test (item 3.12 MDS-UPDRS).

### Ophthalmological assessment

#### Subjective assessment

Some of the ophthalmological assessments require responses from the patient and are therefore defined as subjective measurements. The outcomes are visual acuity, visual field testing, contrast sensitivity, colour vision, ocular motility, facial emotion recognition and the visual function questionnaire (VFQ-25) concerning visual function. We start the assessment with measuring the best corrected visual acuity at distance, by using the Early Treatment of Diabetic Retinopathy Study (ETDRS) chart at 6 m [47]. 20/30–20/60 is considered mild vision loss, or near-normal vision, 20/70–20/160, moderate visual impairment and 20/200–20/400 severe visual impairment. This is followed by testing reading or near visual acuity, tested with the Radner reading charts at 40 cm distance [48]. Reading correction is applied if applicable. The reading speed is scored in words per minute. Possible visual fields deficits including problems in the peripheral vision are tested with the Humphrey or Octopus Automated Field Analyzer in a standardized design (SITA STANDARD 30–2) and analyzed by an ophthalmologist [49]. To measure the ability to detect objects at low contrast, we will measure the contrast sensitivity with the Pelli-Robson charts. This assessment consists of letters arranged in groups with varying contrast, from high to low. Scores are based on the contrast of the last group in which two or three letters were correctly read, this can be calculated in a logarithmic contrast sensitivity score (CSS). In an elderly population (above 60 years old) a CSS lower than 1.50 is considered as decreased contrast sensitivity in [50]. Colour discrimination is the ability to distinguish differences between shades of colours and is divided in primary colours (green, red, blue and yellow) and their axis (red-green, blue-yellow). To evaluate colour vision pseudo-isochromatic plates with coloured dots forming numbers (Ishihara plates) will be used as a screening tool. As a second step the Farnsworth desatured 15D hue test will be performed to evaluate more subtle colour vision deficiencies. This task consists of ordering 15 coloured caps over trays in an incremental manner according to their hue. Colour vision deficiency will be scored in type of colour deficiency and in severity [51, 52].

The examination of ocular motility will be done by an orthoptist. First, we look at eye follow movements, to detect any gaze paresis, nystagmus or saccadic intrusions. Moreover, patients will be asked if they experience diplopia. Diplopia is more common when there is pre-existent ocular misalignment, this is a deviation in the fusion mechanism of the eyes. We use the alternate-cover test at 30 cm and 6 m distance [53] to screen for this. Additionally, convergence insufficiency can cause diplopia and is characterized by an increased near point of convergence (NPC), decreased convergence amplitudes and an exodeviation at near. NPC and exodeviation at near will be measured along a RAF near point rule (Royal Air Force Rule), here we measure if patients can maintain single vision, when trying to focus on an approaching subject. Healthy (young) individuals can avoid double vision until 6 cm in front of their nose. A NPC value that is more than 10 cm from the bridge of the nose is considered abnormal. Convergence amplitudes are measured with a prism, while the patient focuses on a target at near. In general, fusional convergence amplitudes of less than 20 prism diopters at near are a sign of convergence insufficiency [54–56]. In addition, we will use an exploratory Eye-Tracking set up with a Tobii TX300 Eye-Tracker to measure ocular movements and smooth pursuit. Furthermore, we investigate visual and vocal emotion perception and processing using the Emotion Recognition Index (ERI) [57, 58]. This instrument assesses the ability of individuals to correctly infer target emotions from actor portrayals of vocal and facial emotion expressions.

#### Objective assessment

Results of these tests will be obtained without receiving feedback from the patient and are therefore objective measurements. The outcomes are changes in the eyelids, conjunctiva, cornea, fundus, iris and lens opacity, corneal thickness and intraocular pressure, tear production, anatomical changes of the retina including micro vascular changes. First, we examine the eyelids and conjunctiva, followed by inspection of the cornea, iris and using slit lamp examination. This to detect for example blepharitis or conjunctivitis. Lens opacity will be rated with the LOCSIII score, this to evaluate and grade possible cataract [59]. After pupil dilation with tropicamide 0,5% fundus photography is captured. The same experienced ophthalmologist will inspect and grade all fundus images. We will evaluate the macula, retina and vascular structures. This to detect possible presence of macular degeneration, macular edema, vascular pathology, peripapillary atrophy, retinopathy, optic nerve pallor and to evaluate cup-to-disc ratio. A ratio greater than 0.5 may imply glaucoma or other pathology. Furthermore the thickness of the cornea will be measured with pachymetry, this is important because it can mask an accurate reading of the intraocular pressure(IOP). Moreover, a reference range in PD is not known. Pachymetry will be performed after applying local drops of anaesthetics. Corneal thickness ranges between 500 and 575 μm in the healthy population [60]. The IOP will be measured using applanation tonometry (Goldmann tonometer), non-contact tonometry is less reliable [61]. An eye pressure higher than 18mmhg is considered higher than normal and has an increased risk for glaucomatous damage of the optic disc [62]. Complaints of the ocular surface are mainly caused by affections of tear quality and quantity. Tear film quality will be approached by the TearFilmBreakUpTime (TFBUT), while the quantity of tears is will be measured by the Schirmer test. TFBUT is recorded as the time between a complete blink and the appearance of the first randomly distributed dry spot, where a score less than 10 s is considered abnormal [63]. The Schirmer test measures the amount of fluid appearing on the ocular surface within 5 min. For this, paper strips are inserted into the lower fornix with (Schirmer II) and without (Schirmer I) local anaesthesia, and the wet distance is measured in millimetres. Less than 10 mm is considered abnormal and less than 5 is severe [26, 63, 64]. If the Schirmer test and/or TFBUT show lower-than-normal results, the diagnosis “dry eye” or keratoconjunctivitis sicca in case of clinical complaints is made. In Parkinson patients, a reduced eye blink rate EBR may add to an ocular surface disease and dry eye symptoms The EBR is defined as the number of eye blinks per minute measured in a 3-min interval, less than 15 blinks per minute is considered abnormal [26, 65]. Finally, optical coherence tomography (OCT) will be performed to evaluate pattern changes of the retina including the retinal thickness [66–68]. Angio-OCT will be used to inspect the micro vascular changes of the retina [69].

### Statistical analysis plan

95% Confidence intervals, means, standard deviations and frequency distributions will be calculated for all outcomes. Nonparametric variables (age, disease duration, LED, total score VIPD-Q) will be expressed as the median, interquartile range (IQR), minimum, and maximum. To compare groups, we will use the student’s t-test for parametric continuous variables and the Mann-Whitney U test for non-parametric continuous variables. All analyses will be performed with SPSS 22.0 (SPSS Inc., IBM, Chicago, IL, USA).

To address the first study objective (validity of the screening questionnaire), we will compare the results of the VIPD-Q with the ophthalmological assessments using analysis of intraclass correlation coefficient (ICC), with Cohen’s kappa. The results of the ophthalmological assessments will be categorized in visual disorders within the domains of the VIPD-Q (ocular surface, intra- ocular, oculomotor and optic nerve). We hypothesize moderate to good correlation (ICC > 0.5) between the scores on the domains based on the ophthalmological tests and the scores on the domains based on the screening questionnaire. We will also address correlation for the total score. In addition, we will calculate optimal cut-off points of the domains and total score of the VIPD-Q using the receiver operating characteristic (ROC).

For the second and third objective (determine the prevalence and severity of visual problems in PD; impact on clinical functioning), PD patients will be compared with healthy controls using chi-square-tests for categorical values (sex, education, co morbidity, visited ophthalmologist, visual aid, vision changes during the day, difficulties with daily activities, impact on quality of life) or Mann-Whitney U test for non-parametric continuous variables (age, total score VIPD-Q, score per domain). Furthermore, the relationship between falling, disease duration, MDS-UPDRS, hand-eye dexterity, gait and balance tests, and visual problems will be assessed using multiple linear regression analysis.

### Data management

The certified data management system “Castor” will be used Each patient will be coded with a unique patient identification number. Personal patient information (such as name and date of birth) is stored separately from the research data. The key to the code is safeguarded by the coordinating researcher. Research data is stored for 15 years and only accessible for members of the research team.

## Discussion

Numerous visual problems have been reported in patients with PD. However, these problems appear to be markedly under-recognized as well as undertreated, and are also being poorly understood. Patients may not adequately report ophthalmic problems themselves, while clinicians frequently miss ocular disorders that in many cases can be treated. This may result in a delayed diagnosis and further deterioration of visual impairment. Moreover, undertreated patients experience an unnecessarily high risk of falling and sustaining injuries. Previous research has shown contradicting outcomes on the prevalence and suggested pathology of eye problems in PD [2, 11, 13, 70, 71]. The present VIP study is designed to provide new insights into the field of visual function in PD. Our theory-based screening questionnaire is designed to timely detect visual problems and to heighten the awareness of visual impairment in the PD population. It may assist in earlier detection of visual symptoms and thus help clinicians to select optimal diagnostic and therapeutically strategies. This could lead to improved patient care and an improved quality of life for PD patients.

## Trial status

The final protocol version is 3.0 and date April 2017. This trial is currently recruiting participants. Recruitment of phase 1 has started December 2016 and was completed August 2018. Recruitment of phase 2 began in May 2017. We expect the recruitment of phase 2 to be complete by May 2019.

## Additional files

Additional file 1: Screening questionnaire: Visual impairment in Parkinson’s disease. VIPD-Q questionnaire original format. (DOCX 29 kb)

Additional file 2: Medication that influences normal visual function other than PD medication. Commonly used drugs associated with eye diseases, risk 1% or more [73]. (DOCX 12 kb)

## Data Availability

Data sharing is not applicable to this article as no datasets were generated or analysed during the current study.

## Abbreviations

AUC: Area under the curve
CLOX: Clock drawing test
CSS: The logarithmic contrast sensitivity score
D: Dioptre
DM type I: Diabetes type I
EBR: Eye blink rate
ERI: Emotion Recognition Index
ETDRS: Early Treatment of Diabetic Retinopathy Study
FOG: Freezing of gait
GDS: Geriatric depression scale
ICC: Intraclass correlation coefficient
IOP: Intra-ocular pressure
IQR: Interquartile range
LED: Levodopa equivalent dose
MDS-UPDRS: Movement disorders society - Unified Parkinson’s Disease rating scale
MoCA: Montreal Cognitive Assessment
NMSS: Non-motor symptoms assessment scale
NPC: Near point of convergence
OCT: Optical coherence tomography
OLVG: onze lieve vrouwen gasthuis (hospital)
PD: Parkinson’s disease
PDQ-39: the Parkinson’s disease questionnaire-39
RAF: Royal Air Force Rule, near point rule
ROC: Receiver operating characteristic
SLS: Single leg stance test
TFBUT: TearFilmBreakUpTime
TUG: Timed up and go test
VFQ-25: Visual function questionnaire
VIP: Visual impairment in Parkinson’s disease
VIPD-Q: Visual impairment in Parkinson’s disease questionnaire

## References

[1] de Lau LM, Koudstaal PJ, Hofman A, Breteler MM (2006). Subjective complaints precede Parkinson disease: the Rotterdam study. Arch Neurol.

[2] Davidsdottir S, Cronin-Golomb A, Lee A (2005). Visual and spatial symptoms in Parkinson’s disease. Vis Res.

[3] Archibald NK, Clarke MP, Mosimann UP, Burn DJ (2009). The retina in Parkinson’s disease. Brain.

[4] Nguyen-Legros J (1988). Functional neuroarchitecture of the retina: hypothesis on the dysfunction of retinal dopaminergic circuitry in Parkinson’s disease. Surg Radiol Anat.

[5] Pascolini D, Mariotti SP (2012). Global estimates of visual impairment: 2010. Br J Ophthalmol.

[6] Nonnekes J, Nieuwboer A, Hallett M, Fasano A, Bloem B. Compensation strategies for gait impairments in Parkinson’ disease. JAMA Neurol. 2019. 10.1001/jamaneurol.2019.0033 30907948

[7] Azulay JP, Mesure S, Amblard B, Blin O, Sangla I, Pouget J (1999). Visual control of locomotion in Parkinson’s disease. Brain.

[8] Azulay JP, Mesure S, Amblard B, Pouget J (2002). Increased visual dependence in Parkinson’s disease. Percept Mot Skills.

[9] Wood BH, Bilclough JA, Bowron A, Walker RW (2002). Incidence and prediction of falls in Parkinson’s disease: a prospective multidisciplinary study. J Neurol Neurosurg Psychiatry.

[10] Rumalla K, Gondi KT, Reddy AY, Mittal MK (2017). Association of Parkinson’s disease with hospitalization for traumatic brain injury. Int J Neurosci.

[11] Biousse V, Skibell BC, Watts RL, Loupe DN, Drews-Botsch C, Newman NJ (2004). Ophthalmologic features of Parkinson’s disease. Neurology.

[12] Archibald NK, Clarke MP, Mosimann UP, Burn DJ (2011). Visual symptoms in Parkinson’s disease and Parkinson’s disease dementia. Mov Disord.

[13] Nowacka B, Lubinski W, Honczarenko K, Potemkowski A, Safranow K (2014). Ophthalmological features of Parkinson disease. Med Sci Monit.

[14] Diederich NJ, Raman R, Leurgans S, Goetz CG (2002). Progressive worsening of spatial and chromatic processing deficits in Parkinson disease. Arch Neurol.

[15] Matsui H, Udaka F, Tamura A, Oda M, Kubori T, Nishinaka K, Kameyama M (2006). Impaired visual acuity as a risk factor for visual hallucinations in Parkinson’s disease. J Geriatr Psychiatry Neurol.

[16] Ekker MS, Janssen S, Seppi K, Poewe W, de Vries NM, Theelen T, Nonnekes J, Bloem BR. Ocular and visual disorders in Parkinson’s disease: common but frequently overlooked. Parkinsonism Relat Disord. 2017. 10.1016/j.parkreldis.2017.02.014 28284903

[17] Goldberg I, Hollows FC, Kass MA, Becker B (1981). Systemic factors in patients with low-tension glaucoma. Br J Ophthalmol.

[18] Buttner T, Kuhn W, Klotz P, Steinberg R, Voss L, Bulgaru D, Przuntek H (1993). Disturbance of colour perception in Parkinson’s disease. J Neural Transm Park Dis Dement Sect.

[19] Repka MX, Claro MC, Loupe DN, Reich SG (1996). Ocular motility in Parkinson’s disease. J Pediatr Ophthalmol Strabismus.

[20] Muller T, Kuhn W, Buttner T, Przuntek H (1997). Distorted colour discrimination in Parkinson’s disease is related to severity of the disease. Acta Neurol Scand.

[21] Pieri V, Diederich NJ, Raman R, Goetz CG (2000). Decreased color discrimination and contrast sensitivity in Parkinson’s disease. J Neurol Sci.

[22] Hutton JT, Morris JL (2001). Vision in Parkinson’s disease. Adv Neurol.

[23] Muller T, Woitalla D, Peters S, Kohla K, Przuntek H (2002). Progress of visual dysfunction in Parkinson’s disease. Acta Neurol Scand.

[24] Sprengelmeyer R, Young AW, Mahn K, Schroeder U, Woitalla D, Buttner T, Kuhn W, Przuntek H (2003). Facial expression recognition in people with medicated and unmedicated Parkinson’s disease. Neuropsychologia.

[25] Williams DR, Lees AJ (2005). Visual hallucinations in the diagnosis of idiopathic Parkinson’s disease: a retrospective autopsy study. Lancet Neurol.

[26] Tamer C, Melek IM, Duman T, Oksuz H (2005). Tear film tests in Parkinson’s disease patients. Ophthalmology.

[27] Diederich NJ, Fenelon G, Stebbins G, Goetz CG (2009). Hallucinations in Parkinson disease. Nat Rev Neurol.

[28] Seichepine DR, Neargarder S, Miller IN, Riedel TM, Gilmore GC, Cronin-Golomb A (2011). Relation of Parkinson’s disease subtypes to visual activities of daily living. J Int Neuropsychol Soc.

[29] Armstrong RA (2015). Oculo-visual dysfunction in Parkinson’s disease. J Park Dis.

[30] Lin TP, Rigby H, Adler JS, Hentz JG, Balcer LJ, Galetta SL, Devick S, Cronin R, Adler CH (2015). Abnormal visual contrast acuity in Parkinson’s disease. J Park Dis.

[31] MacAskill MR, Anderson TJ (2016). Eye movements in neurodegenerative diseases. Curr Opin Neurol.

[32] Matlach Juliane, Wagner Martin, Malzahn Uwe, Schmidtmann Irene, Steigerwald Frank, Musacchio Thomas, Volkmann Jens, Grehn Franz, Göbel Winfried, Klebe Stephan (2018). Retinal changes in Parkinson's disease and glaucoma. Parkinsonism & Related Disorders.

[33] Ahn J, Lee JY, Kim TW, Yoon EJ, Oh S, Kim YK, Kim JM, Woo SJ, Kim KW, Jeon B (2018). Retinal thinning associates with nigral dopaminergic loss in de novo Parkinson disease. Neurology.

[34] McDowell SA, Harris JP (1997). Visual problems in Parkinson’s disease: a questionnaire survey. Behav Neurol.

[35] Goetz CG, Tilley BC, Shaftman SR, Stebbins GT, Fahn S, Martinez-Martin P, Poewe W, Sampaio C, Stern MB, Dodel R (2008). Movement Disorder Society-sponsored revision of the unified Parkinson’s disease rating scale (MDS-UPDRS): scale presentation and clinimetric testing results. Mov Disord.

[36] Chaudhuri KR, Prieto-Jurcynska C, Naidu Y, Mitra T, Frades-Payo B, Tluk S, Ruessmann A, Odin P, Macphee G, Stocchi F (2010). The nondeclaration of nonmotor symptoms of Parkinson’s disease to health care professionals: an international study using the nonmotor symptoms questionnaire. Mov Disord.

[37] Peto V, Jenkinson C, Fitzpatrick R, Greenhall R (1995). The development and validation of a short measure of functioning and well being for individuals with Parkinson’s disease. Qual Life Res.

[38] Ruzicka E, Krupicka R, Zarubova K, Rusz J, Jech R, Szabo Z (2016). Tests of manual dexterity and speed in Parkinson’s disease: not all measure the same. Parkinsonism Relat Disord.

[39] Bootsma- van der Wiel A, Gussekloo J, de Craen AJM, van Exel E, Bloem BR, Westendorp RG (2003). Single versus dual task walking performance as predictor of falls in the general population of oldest old. Results of the Leiden 85-plus study. J Am Geriatr Soc.

[40] Mak MK, Pang MY (2010). Parkinsonian single fallers versus recurrent fallers: different fall characteristics and clinical features. J Neurol.

[41] Mak MK, Pang MY (2009). Balance confidence and functional mobility are independently associated with falls in people with Parkinson’s disease. J Neurol.

[42] Snijders AH, Nijkrake MJ, Bakker M, Munneke M, Wind C, Bloem BR (2008). Clinimetrics of freezing of gait. Mov Disord.

[43] Borm C, Krismer F, Wenning GK, Seppi K, Poewe W, Pellecchia MT, Barone P, Johnsen EL, Ostergaard K, Gurevich T (2018). Axial motor clues to identify atypical parkinsonism: a multicentre European cohort study. Parkinsonism Relat Disord.

[44] Jacobs JV, Horak FB, Tran VK, Nutt JG (2006). Multiple balance tests improve the assessment of postural stability in subjects with Parkinson’s disease. J Neurol Neurosurg Psychiatry.

[45] Abdo WF, Borm GF, Munneke M, Verbeek MM, Esselink RA, Bloem BR (2006). Ten steps to identify atypical parkinsonism. J Neurol Neurosurg Psychiatry.

[46] Aerts MB, Esselink RA, Abdo WF, Meijer FJ, Drost G, Norgren N, Janssen MJ, Borm GF, Bloem BR, Verbeek MM (2015). Ancillary investigations to diagnose parkinsonism: a prospective clinical study. J Neurol.

[47] Kinyoun J, Barton F, Fisher M, Hubbard L, Aiello L, Ferris F (1989). Detection of diabetic macular edema. Ophthalmoscopy versus photography--early treatment diabetic retinopathy study report number 5. The ETDRS research group. Ophthalmology.

[48] Maaijwee K, Mulder P, Radner W, Van Meurs JC (2008). Reliability testing of the Dutch version of the Radner Reading charts. Optom Vis Sci.

[49] Landers J, Sharma A, Goldberg I, Graham SL (2010). Comparison of visual field sensitivities between the Medmont automated perimeter and the Humphrey field analyser. Clin Exp Ophthalmol.

[50] Mantyjarvi M, Laitinen T (2001). Normal values for the Pelli-Robson contrast sensitivity test. J Cataract Refract Surg.

[51] Farnsworth D (1957). Testing for color deficiency in industry. AMA archives of industrial health.

[52] Hardy LH (1945). Standard illuminants in relation to color-testing procedures. Arch Ophthal.

[53] Thomas R, Braganza A, George T (1996). Practical approach to diagnosis of strabismus. Indian J Ophthalmol.

[54] Shaunak S, O'Sullivan E, Kennard C (1995). Eye movements. J Neurol Neurosurg Psychiatry.

[55] Danchaivijitr C, Kennard C (2004). Diplopia and eye movement disorders. J Neurol Neurosurg Psychiatry.

[56] Convergence Insufficiency Treatment Trial Study G (2008). Randomized clinical trial of treatments for symptomatic convergence insufficiency in children. Arch Ophthalmol.

[57] Argaud S, Verin M, Sauleau P, Grandjean D (2018). Facial emotion recognition in Parkinson's disease: a review and new hypotheses. Mov Disord.

[58] Scherer KR (2011). Assessing the ability to recognize facial and vocal expressions of emotion: construction and validation of the emotion recognition index. J Nonverbal Behav.

[59] Chylack LT, Wolfe JK, Singer DM, Leske MC, Bullimore MA, Bailey IL, Friend J, McCarthy D, Wu SY (1993). The Lens opacities classification system III. The longitudinal study of cataract study group. Arch Ophthalmol.

[60] Feizi S, Jafarinasab MR, Karimian F, Hasanpour H, Masudi A (2014). Central and peripheral corneal thickness measurement in normal and keratoconic eyes using three corneal pachymeters. J Ophthalmic Vis Res.

[61] Zhao D, Guallar E, Gajwani P, Swenor B, Crews J, Saaddine J, Mudie L, Varadaraj V, Friedman DS, Group STGS (2017). Optimizing Glaucoma screening in high-risk population: design and 1-year findings of the screening to prevent (SToP) Glaucoma study. Am J Ophthalmol.

[62] Tanito M, Itai N, Dong J, Ohira A, Chihara E (2003). Correlation between intraocular pressure level and optic disc changes in high-tension glaucoma suspects. Ophthalmology.

[63] Milner MS, Beckman KA, Luchs JI, Allen QB, Awdeh RM, Berdahl J, Boland TS, Buznego C, Gira JP, Goldberg DF (2017). Dysfunctional tear syndrome: dry eye disease and associated tear film disorders - new strategies for diagnosis and treatment. Curr Opin Ophthalmol.

[64] Kwon OY, Kim SH, Kim JH, Kim MH, Ko MK (1994). Schrimer test in Parkinson's disease. J Korean Med Sci.

[65] Zaman ML, Doughty MJ (1997). Some methodological issues in the assessment of the spontaneous eyeblink frequency in man. Ophthalmic Physiol Opt.

[66] Aaker GD, Myung JS, Ehrlich JR, Mohammed M, Henchcliffe C, Kiss S (2010). Detection of retinal changes in Parkinson's disease with spectral-domain optical coherence tomography. Clin Ophthalmol.

[67] Mailankody P, Battu R, Khanna A, Lenka A, Yadav R, Pal PK (2015). Optical coherence tomography as a tool to evaluate retinal changes in Parkinson's disease. Parkinsonism Relat Disord.

[68] Ucak T, Alagoz A, Cakir B, Celik E, Bozkurt E, Alagoz G (2016). Analysis of the retinal nerve fiber and ganglion cell - inner plexiform layer by optical coherence tomography in Parkinson's patients. Parkinsonism Relat Disord.

[69] de Carlo TE, Romano A, Waheed NK, Duker JS (2015). A review of optical coherence tomography angiography (OCTA). Int J Retina Vitreous.

[70] Armstrong RA (2011). Visual symptoms in Parkinson’s disease. Parkinson’s disease.

[71] Sauerbier A, Ray Chaudhuri K (2013). Parkinson’s disease and vision. Basal Ganglia.

[72] Mangione CM, Lee PP, Gutierrez PR, Spritzer K, Berry S, Hays RD (2001). National eye Institute visual function questionnaire field test I: development of the 25-item National eye Institute visual function questionnaire. Arch Ophthalmol.

[73] Santaella RM, Fraunfelder FW (2007). Ocular adverse effects associated with systemic medications : recognition and management. Drugs.

