# Supplementary material for: Towards seeing the visual impairments in Parkinson’s disease: protocol for a multicentre observational, cross-sectional study
Source: BMC Neurol. 2019 Jun 25;19:141. doi: 10.1186/s12883-019-1365-8 (PMC6591947; doi:10.1186/s12883-019-1365-8)
Supplement: Supplementary file 1 — Screening questionnaire: Visual impairment in Parkinson’s disease. VIPD-Q questionnaire original format. (DOCX 29 kb) [file 12883_2019_1365_MOESM1_ESM.docx]

**
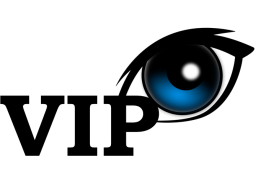
Appendix 1 screening questionnaire: Visual impairment in Parkinson’s disease**Part I: Judge the complaints / problems you have been experiencing the last month including today:

|  | Never | 1-2x/month | Every week | Daily |
| --- | --- | --- | --- | --- |
| 1. I have blurry vision when I read or work on a computer. |  |  |  |  |
| 2. I have a burning sensation or gritty feeling in my eyes. |  |  |  |  |
| 3. I have mucus / slime or particles in my eyes or eyelids. |  |  |  |  |
| 4. I have watery eyes. |  |  |  |  |
| 5. When I read, some letters disappear. |  |  |  |  |
| 6. Lines that should be straight appear to be wavy or blurred. |  |  |  |  |
| 7. I won’t go out alone in the evening or at night because my night vision is insufficient. |  |  |  |  |
| 8. When I drive at night, the oncoming headlights cause more glare than before. |  |  |  |  |
| 9. Quick movements are hard to follow with my eyes. |  |  |  |  |
| 10. I have double vision. |  |  |  |  |
| 11. I can read better with one eye closed. |  |  |  |  |
| 12. I have trouble with depth perception. I find it hard to say which one of two objects is closer. |  |  |  |  |
| 13. Colours seem to be paler than before. |  |  |  |  |
| 14. I can’t read plain text on a coloured or grey background. |  |  |  |  |
| 15. I run into objects or people or feel that parts of my visual field are missing. |  |  |  |  |
| 16. I have problems with rapid changes of light intensity. (For example driving through a tunnel.) |  |  |  |  |
| 17. I see things that other people do not see (hallucinations). |  |  |  |  |
| 18. I have difficulties recognizing faces. |  |  |  |  |
| 19. I have problems reading, because I see letters (too) late/slow. (Therefore I have difficulties reading subtitles on TV or road signs.) |  |  |  |  |
| 20. I see after-images when I look away from an object. |  |  |  |  |
| 21. People’s body parts appear too large or look strange. |  |  |  |  |
| 22. Bright light causes discomfort. I feel the need to squint or close my eyes. |  |  |  |  |

|  | Never | 1-2x/month | Every week | Daily |
| --- | --- | --- | --- | --- |
| Scoring sheet | 0 | 1 | 2 | 3 |
| Maximum reachable points: 66 | | | Minimum reachable points 0 | |
