# Supplementary material for: Towards seeing the visual impairments in Parkinson’s disease: protocol for a multicentre observational, cross-sectional study
Source: BMC Neurol. 2019 Jun 25;19:141. doi: 10.1186/s12883-019-1365-8 (PMC6591947; doi:10.1186/s12883-019-1365-8)
Supplement: Supplementary file 2 — Medication that influences normal visual function other than PD medication. Commonly used drugs associated with eye diseases, risk 1% or more [73]. (DOCX 12 kb) [file 12883_2019_1365_MOESM2_ESM.docx]

**Appendix 2: Medication that influences normal visual function other than PD medication**

| Generic Name |
| --- |
| Paclitaxel |
| Systemic and topic steroids (around the eyes) |
| Fluocinolone |
| Fluocinonide |
| Thioridazine |
| Triamcinolone (injectable) |
| Triamcinolone (topical) |
| Chloroquine |
| Hydroxychloroquine |
| Isotretinoin |
| Tamoxifen |
| Tretinoin |
| Amiodarone |
| Ethambutol |
| Linezolid |
| Sildenafil |
| Tadalafil |
| Vardenafil |

Commonly used drugs associated with eye diseases, risk 1% or more[73].
